# The association between sugar-sweetened beverage consumption, muscle strength, and psychological symptoms among Chinese adolescents: a multicenter cross-sectional survey

**DOI:** 10.3389/fnut.2025.1641108

**Published:** 2025-07-31

**Authors:** Yanjie Zhou, Chunhua Xue, Gulnur Ahmat, Huijuan Lou, Yun Liu, Li Ma

**Affiliations:** ^1^Henan Vocational College of Agriculture Public Sports College, Zhengzhou, China; ^2^Sports Art Education Teaching and Research Department of University of International Relations, Beijing, China; ^3^Business School, Xinjiang Normal University, Ürümqi, Xinjiang, China; ^4^School of Physical Education, Chizhou University, Chizhou, China

**Keywords:** sugar-sweetened beverage consumption, muscle strength, psychological symptoms, association, adolescents

## Abstract

**Background:**

The prevalence of psychological symptoms in adolescents has been increasing and has become an important public health issue of concern to countries around the world. However, no studies have been conducted on the association between sugar-sweetened beverage (SSB) consumption, muscle strength, and psychological symptoms in Chinese adolescents. The present study may provide theoretical support and assistance for the prevention and intervention of psychological symptoms in Chinese adolescents.

**Methods:**

In this study, 42,832 adolescents aged 12–17 years in mainland China were assessed cross-sectionally for SSB consumption, standing long jump reflecting muscle strength, psychological symptoms, and related covariates using a three-stage stratified whole-cluster random sampling method. The methods of univariate analysis, logistic regression analysis, and binary Logistic regression analysis with a generalized linear model were used for the analysis.

**Results:**

The prevalence of psychological symptoms among Chinese adolescents aged 12–17 years was 21.2%; the prevalence of boys (22.0%) was higher than that of girls (20.3%), and the difference was statistically significant (*χ*^2^ = 18.320, *p* < 0.001). The percentage of adolescents with SSB consumption frequency of ≥4 times/week was 14.6%. The mean standing long jump of adolescents was (186.80 ± 33.16) cm. Binary Logistic regression analysis with the generalized linear model was performed with the presence of psychological symptoms in adolescents as the dependent variable and different combinations of SSB consumption and standing long jump quartile as independent variables. Overall results showed that the risk of psychological symptoms among adolescents in the SSB consumption ≥4 times/week and standing long jump quartile Q1 group was higher than that of adolescents in the SSB consumption ≤1 times/week and standing long jump quartile Q4 group adolescents by 2.05 times (95% CI: 1.76–2.38) (*p* < 0.001).

**Conclusion:**

There is an association between SSB consumption, muscle strength, and psychological symptoms in Chinese adolescents. Effective reduction of SSB consumption and improvement of muscle strength may be an effective way to reduce psychological symptoms. The effects of SSB consumption and muscle strength should be emphasized in the prevention and intervention of adolescents’ psychological symptoms in the future.

## 1 Introduction

The changes in modern lifestyles have led to the emergence of irregular eating behaviors, prolonged screen time, decreased levels of physical activity, prolonged static behaviors, and increased rates of obesity among adolescents (1). The emergence of these maladaptive problems is a significant contributor to the continued increase in the prevalence of psychological symptoms in adolescents, with important negative effects on adolescents’ academic performance, employment, and future achievement (2). A survey by the World Health Organization shows that in 2021, about 280 million people around the world had have varying degrees of psychological symptoms, accounting for 3.8% of the total global population, of which the mental health of the youth population is not optimistic (3). It has also been shown that the global prevalence of psychological symptoms increased 1.8-fold from 1990 to 2021 and continues to trend higher, placing a serious burden of disease on countries (4). Another survey of U. S. adolescents from 2013 to 2023 also showed that more than 5.3 million adolescents aged 12–17 have varying degrees of psychological symptoms, and the prevalence is continuing to rise, posing a serious threat to adolescent health (5). A survey of Chinese adolescents found that the prevalence of psychological symptoms among Chinese adolescents rose from 18 to 24% from 2004 to 2019, with more significant increases among girls and urban adolescents, which should cause some concern (6). Another study on Chinese adolescents also showed that the prevalence of psychological symptoms among adolescents increased from 15.4% in 2000 to 28.3% in 2020, and continues to show an increasing trend (7). Research has shown that the development of psychological symptoms in adolescence can have a profound impact on future adult health, leading to an increased risk of developing various types of mental illnesses in adulthood (8). Prevention and intervention of psychological symptoms in adolescence will have a significant impact on adolescent health and future adult development. However, the factors that contribute to the increased prevalence of psychological symptoms in adolescents are multiple (9). Past research has focused on physical exercise, sleep quality, and physical activity, and less research has been conducted on the increased SSB consumption and decreased muscle strength associated with psychological symptoms that are particularly prominent in current adolescents.

Sugar-sweetened beverage consumption is rising globally and poses a serious threat to adolescent health (10). Studies show that SSB consumption among adolescents has increased by an average of 50–100 mL per day globally over the past decade from 2010 to 2020, and continues to rise in low- and middle-income countries, including China, posing a serious threat to adolescent health (11). Another survey of Chinese adolescents also showed that the average daily SSB consumption of Chinese adolescents increased from 200 mL in 2011 to 350 mL in 2021, with more significant increases in urban areas (12). Several studies have confirmed that SSB consumption overdose in adolescents is an important risk factor for cardiovascular disease and increased risk of all-cause mortality, and should be given adequate attention and concern (13). In addition, it has been found that increased SSB consumption leads to the development of various psychological disorders, such as depression, anxiety, and other psychological problems that show a trend of increased incidence (14). It has also been shown that increased SSB consumption leads to increased rates of obesity, which can also hurt mental health (15). However, past research has also found that SSB consumption leads to the secretion of dopamine in the organism, which has some positive effects on the development of mental health (16–18). Unfortunately, few studies have been conducted on the association between SSB consumption and psychological symptoms in Chinese adolescents. Therefore, analyzing the association between SSB consumption and psychological symptoms using a nationally representative sample of Chinese adolescents will play a positive role in the prevention of psychological symptoms in adolescents.

There is a strong correlation between adolescent muscle strength and health. The study found that there is an association between muscle strength and mental health in adolescents and that maintaining a certain level of muscle strength positively affects an individual’s outward appearance and has beneficial effects on mental health (19). However, research in recent years has found that the muscle strength of adolescents in many countries around the world, including Chinese adolescents, is showing a downward trend, which has a certain impact on physical and mental health. Studies have shown that the muscle strength of 12–15-year-olds in the United States has shown a declining trend, with an average decrease of 12–15 cm in standing long jump performance between 1990 and 2020 (20). Another study that included adolescents in 17 countries around the world found that adolescents’ standing long jump performance declined by an average of 8–10% over the past 30 years (21). However, Chinese adolescents are no exception. Surveys show that the standing long jump, which reflects the muscular strength of Chinese adolescents, has decreased from 215 cm in 2010 to 208 cm in 2020, a decrease of 3.3% on average, which hurts adolescents’ physical health (22). Studies have found that a decline in muscle strength in adolescents has an impact leading to lower levels of physical fitness, which in turn affects levels of mental health (1). It has also been shown that declines in muscle strength are often accompanied by changes in cognitive functioning of the brain, brain activation states, and some negative effects on mental health (23). This shows that it is important for adolescents to maintain a certain level of muscle strength for the development of mental health.

In conclusion, adolescent SSB consumption and muscle strength have some impact on adolescent mental health. Although the results are not unanimous there is even some disagreement. Therefore, it is worthwhile to further explore and analyze this issue. However, with the continuous increase of psychological symptoms among Chinese adolescents, it is necessary to investigate the association between SSB consumption and muscle strength with psychological symptoms among Chinese adolescents. Unfortunately, no studies have been found on the association between the combined effects of SSB consumption and muscle strength on psychological symptoms in Chinese adolescents. For this reason, the present study was a cross-sectional investigation of SSB consumption, muscle strength, and psychological symptoms among 42,832 adolescents in mainland China. The aim was to analyze the associations that exist between SSB consumption, muscle strength, and psychological symptoms in Chinese adolescents. This study will provide theoretical support and assistance for the prevention and intervention of psychological symptoms in Chinese adolescents.

## 2 Methods

### 2.1 Participants

**Figure 1:**
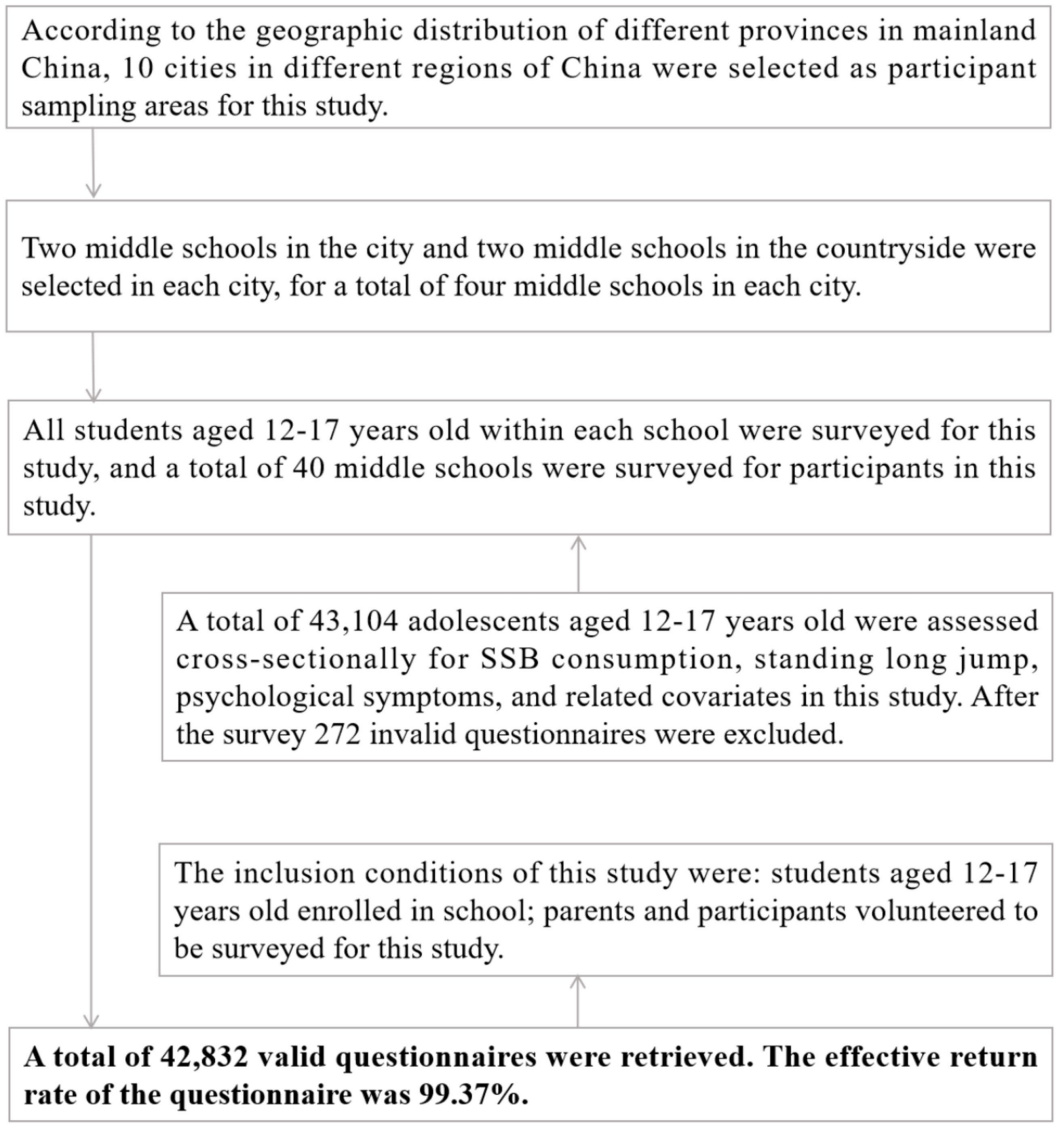
Extraction process for Chinese youth participants aged 12–17 years old.

In this study, a three-stage stratified whole cluster random sampling method was used to select participants. In the first stage, according to the geographic distribution of different provinces in mainland China, 10 cities in different regions of China were selected as participant sampling areas for this study. Changchun City and Jilin City in Jilin Province in the northern region of China; Guangzhou City and Zhuhai City in Guangdong Province in the southern region of China; Hefei City and Huangshan City in Anhui Province in the eastern region of China; Zhengzhou City and Nanyang City in Henan Province in the central region of China; and Xi’an City; Xianyang City in Shaanxi Province in the western region of China, were selected, respectively. In the second stage, two middle schools in the city and two middle schools in the countryside were selected in each city, for a total of four middle schools. In the third stage, all students aged 12–17 years old within each school were surveyed for this study, and a total of 40 middle schools were surveyed for participants in this study. The inclusion conditions of this study were: students aged 12–17 years old enrolled in school; parents and participants volunteered to be surveyed for this study. A total of 43,104 adolescents aged 12–17 years old were assessed cross-sectionally for SSB consumption, standing long jump, psychological symptoms, and related covariates in this study. After the survey, 272 invalid questionnaires were excluded. The effective return rate of the questionnaire was 99.37%. The specific extraction process of the participants is shown in Figure 1.

This study was done in keeping with the Declaration of Helsinki. Written assent was obtained from parents/guardians. The participants also signed up for the study on their own. The Ethics Committee of Chizhou University (20246423451) gave the green light for this study.

### 2.2 Assessment of psychological symptoms

In this study, the Multidimensional Sub-health Questionnaire for Adolescents (MSQA), developed by Prof. Fangbiao Tao’s team, was used as an assessment tool for psychological symptoms (23). The questionnaire was used for data collection through participant self-report. The questionnaire has been widely used among Chinese adolescents. The questionnaire consists of 39 questions assessing the participants’ psychological symptoms in the past 6 months, each of which provides 6 options corresponding to different symptom durations: level 6 (“none or less than 1 week”), level 5 (“1–2 weeks”), level 4 (“3–4 weeks”), level 3 (“1–3 months”), level 2 (“2–3 months”), and level 1 (“lasted more than 3 months”), and Level 1 (“lasted more than 3 months”). For scoring purposes, choices of levels 6, 5, and 4 were scored as 0, and choices of levels 3, 2, and 1 were scored as 1. The questionnaire contains three core dimensions, namely emotional problems, behavioral problems, and social adjustment problems. The criteria for positive determination of each dimension were as follows: scores of ≥3 for the dimension of emotional problems, ≥1 for the dimension of behavioral problems, and ≥4 for the dimension of social adjustment problems. When the total score of the three dimensions reached ≥8, the participant was judged to have psychological symptoms. The MSQA scale has good psychometric properties, and its internal consistency reliability Cronbach’s *α* coefficient is 0.854, indicating that the instrument has high reliability (24). The questionnaire has been widely used in Chinese adolescent research, and its validity has been verified by empirical studies to effectively assess the psychological symptoms of the Chinese adolescent population (25–27).

### 2.3 Assessment of SSB consumption

The beverage intake questionnaire (BEVQ-15) is a standardized assessment tool currently in use internationally (28). The questionnaire was developed by a team from Virginia Tech and was specifically designed to quickly assess participants’ daily beverage intake patterns, with a particular focus on SSB consumption. The questionnaire consisted of 15 items, including regular carbonated beverages, sports drinks, sweetened teas, juice drinks, and other sugary beverages, as well as beverages from the healthier drinks category, such as sugar-free beverages, pure fruit juices, cow’s milk, plant-based milk, and drinking water. A self-assessment questionnaire format was used, in which participants were asked to recall the frequency of consumption of each type of SSB and the amount consumed in a single sitting (using a standardized container of 330 mL cans as a reference) in the past 1 month. The BEVQ-15 was used to calculate the total daily intake by converting the frequency option into the average daily intake and combining it with the single serving size. The questionnaire has good psychometric properties with Cronbach’s alpha coefficients of 0.79–0.86. Scale validity with the 24-h dietary recall method was as high as *r* = 0.72 (*p* < 0.01) (29). Despite the limitation of possible underestimation of actual intake (due to reliance on participant memory accuracy), its simplicity and short time consumption (5–10 min) make it an ideal tool for large-scale epidemiologic surveys and public health studies. Taking into account the practicalities of assessment in this study, SSB consumption was categorized as ≤1 time/week, 2–3 times/week, and ≥4 times/week. 330 mL cans were used as the standard for each assessment in this study.

### 2.4 Assessment of muscle strength

In this study, the standing long jump performance was used to indirectly reflect the participants’ muscle strength level. The assessment method was based on the instruments and methods required by the China National Survey on Students’ Constitution and Health (CNSSCH) (30). Specific assessment method: During the test, participants are required to wear sports shoes and stand behind the starting line with their feet naturally separated, without their toes stepping on the line. Before jumping, 2–3 preparatory arm swings are allowed. Both feet must be off the ground at the same time when jumping, keep the body extended during the air phase, and both feet must land at the same time when landing, and there must be no backward movement. Measure the vertical distance from the front of the jump line to the point where the body most recently landed. The results were evaluated to the nearest 0.1 centimeter. Each participant is given two attempts to jump, and the best performance is recorded. The test site requires a flat, non-slip hard surface. The assessment of standing long jump is supervised by a trained physical education teacher to ensure the accuracy of the data and the safety of the assessment.

### 2.5 Covariates

The covariates in this study included body mass index (BMI), family financial income, commuting method to and from school, snacks, and sleep duration. BMI was calculated based on the participant’s height and weight, and the formula was weight (kg)/height (m^2^). BMI was calculated based on the assessment of participants’ height and weight, and the formula was weight (kg)/height (m^2^). Family financial income was converted to a score based on a self-assessment questionnaire completed by the participants. In this study, family financial income was categorized as <1,000 yuan/month, 1,000–5,000 yuan/month, 5,001–8,000 yuan/month, and >8,000 yuan/month. The commuting method to and from school was categorized as Positive Commuting and Negative Commuting. Positive Commuting includes walking and cycling, while Negative Commuting includes taking busses, private cars, electric scooters, and subways. Snacks were categorized as ≤1 time/week, 2–3 times/week, and ≥4 times/week based on participants’ assessment of the past 7 days. Sleep duration was categorized as <7 h/day, 7–8 h/day, and ≥8 h/day, based on the participants’ calculated average of the time they spent going to sleep and waking up in the past 7 days.

### 2.6 Statistical analysis

The data entry and analysis of this study were carried out using SPSS 25.0 software. Continuous variables were expressed as mean ± SD. Categorical variables were expressed as frequency (percentage). Comparisons between groups of adolescents of different genders and the presence of psychological symptoms were performed using independent samples t-test for continuous variables and chi-square test for categorical variables. In the present study, the prevalence of psychological symptoms was compared between adolescents in different SSB consumption and standing long jump quartiles using a chi-square test. Where standing long jump was stratified into four quartiles Q1–Q4 by age and sex. In terms of association analysis, two methods were used in this study: (1) binary logistic regression analysis with three stepwise-adjusted models: model 1 (unadjusted); model 2 (adjusted for age, BMI, and family financial income); and model 3 (further adjusted for commuting methods to and from school, snacks, sleep duration); (2) Binary Logistic regression analyses of generalized linear models, uniformly adjusted for all the above covariates. All statistical analyses were performed using two-sided tests, with the significance level set at *p* < 0.05.

## 3 Results

In this study, 42,832 Chinese adolescents aged 12–17 years (21,355 boys, 49.86%) were assessed for SSB consumption, standing long jump, and psychological symptoms. The baseline characterization of the Chinese adolescent participants is shown in Table 1. The results of the study showed that the prevalence of psychological symptoms among Chinese adolescents was 21.2%.; the prevalence of boys (22.0%) was higher than that of girls (20.3%), which was statistically significant (*χ*^2^ = 18.320, *p* < 0.001). The prevalence rates of emotional problems, behavioral problems, and social adjustment difficulties were 27.6, 26.7, and 17.4%, respectively. Among Chinese adolescents, the proportions of SSB consumption frequency of ≤1 time/week, 2–3 times/week, and ≥4 times/week were 33.4, 52.0, and 14.6%, respectively. The proportion of boys with SSB consumption frequency ≥4 times/week (17.5%) was higher than that of girls (11.7%), and the difference was statistically significant (*χ*^2^ = 476.420, *p* < 0.001). The mean standing long jump of adolescents was (186.80 ± 33.16) cm; the mean standing long jump of boys (205.62 ± 32.23) cm was higher than that of girls (168.09 ± 21.4) cm and the difference was statistically significant (*t* = 142.008, *p* < 0.001).

**Table 1:** Basic characteristics of participants aged 12–17 in China.

Variable	Boys	Girls	Total	*χ*^2^/*t*-value	*p*-value
*N*	21,355(49.86)	21,477(50.14)	42,832		
Age (years)	14.64 ± 1.62	14.70 ± 1.65	14.67 ± 1.64	−4.013	<0.001
Height (cm)	169.27 ± 9.25	160.94 ± 6.46	165.09 ± 9.00	108.123	<0.001
Weight (kg)	58.88 ± 13.42	51.20 ± 9.27	55.03 ± 12.15	68.898	<0.001
BMI (kg/m^2^)	20.40 ± 3.70	19.73 ± 3.17	20.06 ± 3.46	20.362	<0.001
Standing long jump (cm)	205.62 ± 32.23	168.09 ± 21.4	186.80 ± 33.16	142.008	<0.001
Family financial income				177.405	<0.001
<1,000 yuan/month	2,289(10.7)	2,478(11.5)	4,767(11.1)		
1,000–5,000 yuan/month	7,149(33.5)	8,137(37.9)	15,286(35.7)		
5,001–8,000 yuan/month	6,474(30.3)	6,443(30.0)	12,917(30.2)		
>8,000 yuan/month	5,443(25.5)	4,419(20.6)	9,862(23.0)		
Commuting method to and from school				155.449	<0.001
Positive Commuting	10,018(46.9)	8,791(40.9)	18,809(43.9)		
Negative Commuting	11,337(53.1)	12,686(59.1)	24,023(56.1)		
Snacks				553.998	<0.001
≤1 times/week	4,859(22.8)	6,442(30.0)	11,301(26.4)		
2–3 times/week	12,000(56.2)	12,109(56.4)	24,109(56.3)		
≥4 times/week	4,496(21.1)	2,926(13.6)	7,422(17.3)		
Sleep duration				86.214	<0.001
<7 h/day	3,279(15.4)	3,536(16.5)	6,815(15.9)		
7–8 h/day	14,619(68.5)	15,136(70.5)	29,755(69.5)		
≥8 h/day	3,457(16.2)	2,805(13.1)	6,262(14.6)		
SSB consumption				476.420	<0.001
≤1 times/week	6,251(29.3)	8,071(37.6)	14,322(33.4)		
2–3 times/week	11,376(53.3)	10,889(50.7)	22,265(52.0)		
≥4 times/week	3,728(17.5)	2,517(11.7)	6,245(14.6)		
Standing long jump quartile				14580.347	<0.001
Q1	2084(9.8)	8,669(40.4)	10,753(25.1)		
Q2	3,058(14.3)	7,842(36.5)	10,900(25.4)		
Q3	6,894(32.3)	4,470(20.8)	11,364(26.5)		
Q4	9,319(43.6)	496(2.3)	9,815(22.9)		
Emotional problems	5,929(27.8)	5,878(27.4)	11,807(27.6)	0.837	0.360
Behavioral problems	6,020(28.2)	5,397(25.1)	11,417(26.7)	51.316	<0.001
Social adjustment problems	3,918(18.3)	3,549(16.5)	7,467(17.4)	24.705	<0.001
Psychological symptoms	4,700(22.0)	4,364(20.3)	9,064(21.2)	18.320	<0.001

*N*, numbers; M, Mean; SD, standard deviation; BMI, body mass index; SSB, sugar-sweetened beverage.

Table 2 shows a comparison of the presence or absence of psychological symptoms among different categories of adolescents aged 12–17 in China. The results show that 78.8% of Chinese adolescents aged 12–17 years old do not have problems with psychological symptoms, i.e., they are in a healthy state. The results of this study also showed that the prevalence of psychological symptoms was higher in different sex, family financial income, commuting method to and from school, snacks, sleep duration, SSB consumption, standing long jump quartile Aspects, the prevalence of psychological symptoms were compared and the difference was statistically significant (*χ*^2^ = 18.320, 109.271, 4.532, 222.492, 646.476, 239.275, 18.183, *p* < 0.05).

**Table 2:** Comparison of the presence of psychological symptoms among different categories of adolescents aged 12–17 years in China.

Variable	Psychological symptoms [*N* (%)]		*χ*^2^/*t*-value	*P*-value
	No	Yes		
*N*	33,768(78.8)	9,064(21.2)		
Sex			18.320	<0.001
Boys	16,655(78.0)	4,700(22.0)		
Girls	17,113(79.7)	4,364(20.3)		
Family financial income			109.271	<0.001
<1,000 yuan/month	3,487(73.1)	1,280(26.9)		
1,000–5,000 yuan/month	12,151(79.5)	3,135(20.5)		
5,001–8,000 yuan/month	10,350(80.1)	2,567(19.9)		
>8,000 yuan/month	7,780(78.9)	2082(21.1)		
Commuting method to and from school			4.532	0.033
Positive way	14,918(79.3)	3,891(20.7)		
Negative way	18,850(78.5)	5,173(21.5)		
Snacks			222.492	<0.001
≤1 times/week	5,970(80.4)	1,452(19.6)		
2–3 times/week	19,444(80.7)	4,665(19.3)		
≥4 times/week	8,354(73.9)	2,947(26.1)		
Sleep duration			646.476	<0.001
<7 h/day	4,619(67.8)	2,196(32.2)		
7–8 h/day	23,869(80.2)	5,886(19.8)		
≥8 h/day	5,280(84.3)	982(15.7)		
SSB consumption			239.275	<0.001
≤1 times/week	11,538(80.6)	2,784(19.4)		
2–3 times/week	17,765(79.8)	4,500(20.2)		
≥4 times/week	4,465(71.5)	1780(28.5)		
Standing long jump quartile			18.183	<0.001
Q1	8,323(77.4)	2,430(22.6)		
Q2	8,633(79.2)	2,267(20.8)		
Q3	9,005(79.2)	2,359(20.8)		
Q4	7,807(79.5)	2008(20.5)		

N, numbers; SSB, sugar-sweetened beverage.

Table 3 shows a one-way comparison of SSB consumption, standing long jump, and psychological symptoms among Chinese adolescents aged 12–17 years. The overall results showed that the prevalence of emotional problems, behavioral problems, social adjustment problems, and psychological symptoms among Chinese adolescents aged 12–17 years with different SSB consumption were compared with each other, and the differences were statistically significant (X2 = 224,281, 236,488, and 236,488). The differences were statistically significant (*χ*^2^ = 224.281, 236.488, 254.796, 239.275, *p* < 0.001). In terms of standing long jump quartile, the differences in the prevalence of emotional problems, behavioral problems, social adjustment problems, and psychological symptoms were also statistically significant (*χ*^2^ = 224.281, 23,688, 25,496, 23,975, *p* < 0.001) were statistically significant (*χ*^2^ = 33.674, 31.929, 17.137, 18.183, *p* < 0.01). Overall, the prevalence of psychological symptoms in adolescents tended to increase with the increase in SSB Consumption and the decrease in standing long jump performance.

**Table 3:** A one-way comparison of SSB consumption, standing long jump, and psychological symptoms in Chinese adolescents aged 12–17 years old.

Variable	*N*	Emotional problems			Behavioral problems			Social adjustment problems			Psychological symptoms		
		*N* (%)	*χ*^2^-value	*P-*value	*N* (%)	*χ*^2^-value	*P*-value	*N* (%)	*χ*^2^-value	*P*-value	*N* (%)	*χ*^2^-value	*P*-value
Boys													
SSB Consumption			116.03	<0.001		98.793	<0.001		121.259	<0.001		108.935	<0.001
≤1 times/week	6,251	1,662(26.6)			1,675(26.8)			1,093(17.5)			1,306(20.9)		
2–3 times/week	11,376	2,965(26.1)			3,046(26.8)			1906(16.8)			2,334(20.5)		
≥4 times/week	3,728	1,302(34.9)			1,299(34.8)			919(24.7)			1,060(28.4)		
Standing long jump quartile			17.325	0.001		44.568	<0.001		8.08	0.044		28.7	<0.001
Q1	2084	645(31.0)			668(32.1)			402(19.3)			527(25.3)		
Q2	3,058	839(27.4)			862(28.2)			508(16.6)			730(23.9)		
Q3	6,894	1957(28.4)			2057(29.8)			1,291(18.7)			1,515(22.0)		
Q4	9,319	2,488(26.7)			2,433(26.1)			1717(18.4)			1928(20.7)		
Girls													
SSB Consumption			115.126	<0.001		126.504	<0.001		126.427	<0.001		128.047	<0.001
≤1 times/week	8,071	2024(25.1)			1867(23.1)			1,225(15.2)			1,478(18.3)		
2–3 times/week	10,889	2,949(27.1)			2,672(24.5)			1712(15.7)			2,166(19.9)		
≥4 times/week	2,517	905(36.0)			858(34.1)			612(24.3)			720(28.6)		
Standing long jump quartile			26.858	<0.001		26.377	<0.001		9.472	0.024		27.865	<0.001
Q1	8,669	2,533(29.2)			2,325(26.8)			1,506(17.4)			1903(22.0)		
Q2	7,842	2040(26.0)			1873(23.9)			1,255(16.0)			1,537(19.6)		
Q3	4,470	1,187(26.6)			1,098(24.6)			720(16.1)			844(18.9)		
Q4	496	118(23.8)			101(20.4)			68(13.7)			80(16.1)		
Total													
SSB Consumption			224.281	<0.001		236.488	<0.001		254.796	<0.001		239.275	<0.001
≤1 times/week	14,322	3,686(25.7)			3,542(24.7)			2,318(16.2)			2,784(19.4)		
2–3 times/week	22,265	5,914(26.6)			5,718(25.7)			3,618(16.2)			4,500(20.2)		
≥4 times/week	6,245	2,207(35.3)			2,157(34.5)			1,531(24.5)			1780(28.5)		
Standing long jump quartile			33.674	<0.001		31.929	<0.001		17.137	0.001		18.183	<0.001
Q1	10,753	3,178(29.6)			2,993(27.8)			1908(17.7)			2,430(22.6)		
Q2	10,900	2,879(26.4)			2,735(25.1)			1763(16.2)			2,267(20.8)		
Q3	11,364	3,144(27.7)			3,155(27.8)			2011(17.7)			2,359(20.8)		
Q4	9,815	2,606(26.6)			2,534(25.8)			1785(18.2)			2008(20.5)		

N, numbers; SSB, sugar-sweetened beverage.

Table 4 shows the binary logistic regression analysis of SSB consumption, standing long jump, and psychological symptoms in Chinese adolescents aged 12–17 years. The presence of psychological symptoms in adolescents was used as the dependent variable. Binary logistic regression analyses were performed with adolescents’ SSB consumption and standing long jump quartile as independent variables, respectively. Model 1 was the crude model, Model 2 adjusted age, BMI, and family financial income based on Model 1, and Model 3 adjusted commuting method to and from school, snacks, and sleep duration based on Model 2 duration. Overall the results showed that after adjusting for the covariates of interest and analyzing them with adolescent SSB consumption ≤1 times/week as the reference group, adolescents in the SSB consumption ≥4 times/week group had a higher risk of developing psychological symptoms (OR = 1.45, 95% CI: 1.35 to 1.56) (*p* < 0.001). Similarly, adolescents in the standing long jump quartile Q1 group had 1.09 times (95% CI, 1.01 ~ 1.17) higher risk of psychological symptoms than adolescents in the standing long jump quartile Q4 group (*p* < 0.05). Binary logistic regression analyses after stratification by sex showed the same trend for boys and girls.

**Table 4:** Binary logistic regression analysis of SSB consumption, standing long jump, and psychological symptoms in Chinese adolescents aged 12–17 years old.

Sex/variable	Grouping	Psychological symptoms					
		Model 1		Model 2		Model 3	
		OR (95% CI)	*P-*value	OR (95% CI)	*P-*value	OR (95% CI)	*P-*value
Boys							
SSB consumption	≤1 times/week	1.00		1.00		1.00	
	2–3 times/week	0.98(0.91 ~ 1.06)	0.555	0.99(0.91 ~ 1.06)	0.704	0.94(0.87 ~ 1.02)	0.131
	≥4 times/week	1.50(1.37 ~ 1.65)	<0.001	1.54(1.40 ~ 1.70)	<0.001	1.32(1.19 ~ 1.47)	<0.001
Standing long jump quartile	Q4	1.00		1.00		1.21	
	Q3	1.08(1.00 ~ 1.17)	0.048	1.10(1.02 ~ 1.20)	0.015	1.11(1.03 ~ 1.20)	0.01
	Q2	1.20(1.09 ~ 1.33)	<0.001	1.25(1.13 ~ 1.39)	<0.001	1.26(1.13 ~ 1.41)	<0.001
	Q1	1.30(1.16 ~ 1.45)	<0.001	1.34(1.18 ~ 1.52)	<0.001	1.37(1.21 ~ 1.56)	<0.001
Girls							
SSB consumption	≤1 times/week	1.00		1.00		1.00	
	2–3 times/week	1.11(1.03 ~ 1.19)	0.006	1.11(1.03 ~ 1.20)	0.005	1.03(0.96 ~ 1.12)	0.387
	≥4 times/week	1.79(1.61 ~ 1.98)	<0.001	1.83(1.65 ~ 2.02)	<0.001	1.52(1.37 ~ 1.70)	<0.001
Standing long jump quartile	Q4	1.00		1.00		1.00	
	Q3	1.21(0.94 ~ 1.56)	0.136	1.22(0.95 ~ 1.57)	0.122	1.20(0.93 ~ 1.55)	0.154
	Q2	1.27(0.99 ~ 1.62)	0.059	1.27(0.99 ~ 1.63)	0.056	1.24(0.97 ~ 1.59)	0.092
	Q1	1.46(1.15 ~ 1.87)	0.002	1.45(1.14 ~ 1.86)	0.003	1.45(1.14 ~ 1.86)	0.003
Total							
SSB consumption	≤1 times/week	1.00		1.00		1.00	
	2–3 times/week	1.05(1.00 ~ 1.11)	0.071	1.06(1.00 ~ 1.11)	0.048	1.00(0.95 ~ 1.06)	0.899
	≥4 times/week	1.65(1.54 ~ 1.77)	<0.001	1.69(1.57 ~ 1.81)	<0.001	1.45(1.35 ~ 1.56)	<0.001
Standing long jump quartile	Q4	1.00		1.00		1.00	
	Q3	1.02(0.95 ~ 1.09)	0.590	1.02(0.95 ~ 1.09)	0.606	1.00(0.93 ~ 1.07)	0.952
	Q2	1.02(0.95 ~ 1.09)	0.546	1.02(0.95 ~ 1.10)	0.536	0.97(0.91 ~ 1.04)	0.452
	Q1	1.14(1.06 ~ 1.21)	<0.001	1.12(1.05 ~ 1.21)	0.001	1.09(1.01 ~ 1.17)	0.019

SSB, sugar-sweetened beverage; OR, Odds Ratio; 95% CI, 95% Confidence Interval. Model 1 is the crude model, Model 2 adjusts age, BMI, and family financial income based on Model 1, and Model 3 adjusts commuting method to and from school, snacks, and sleep based on Model 2 duration.

**Figure 2:**
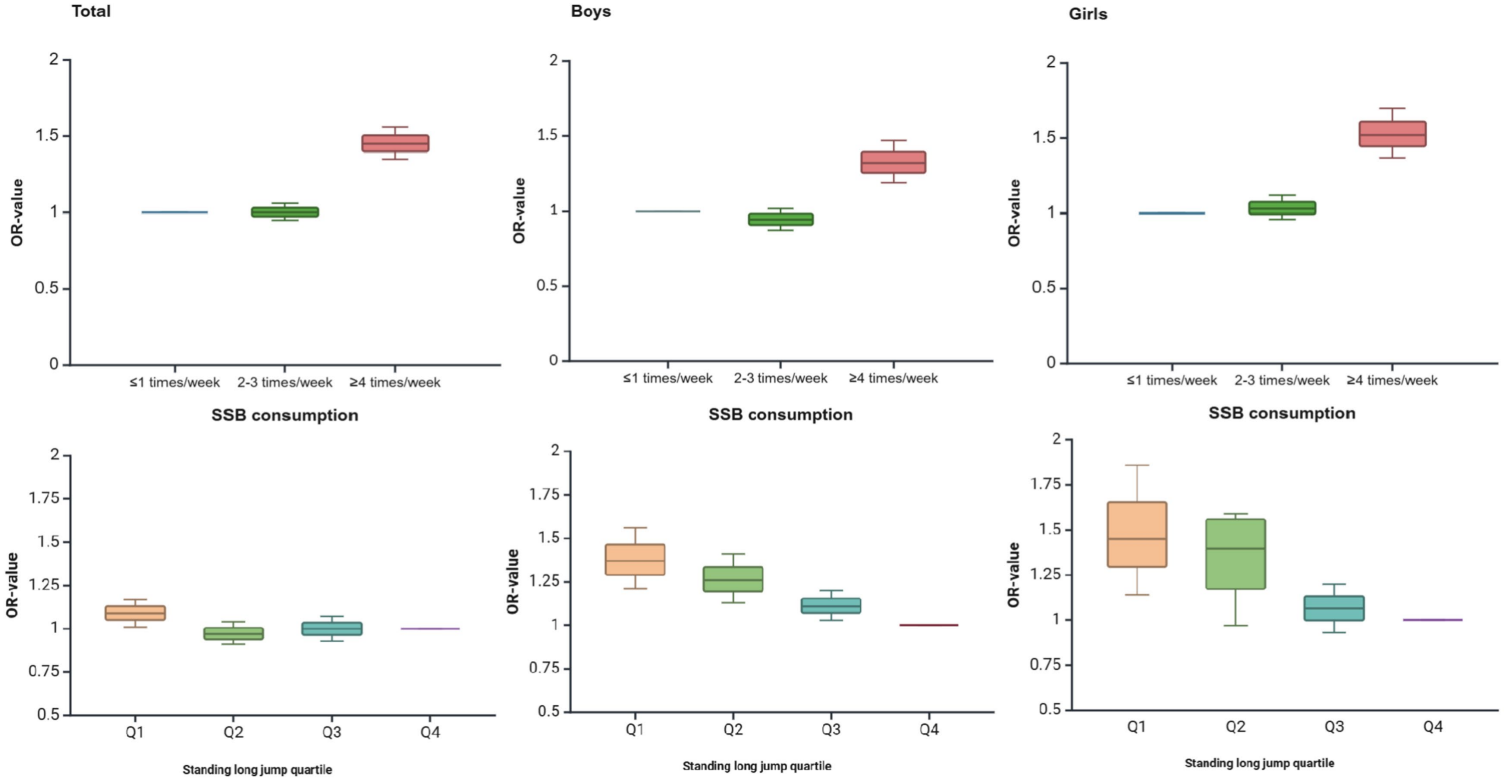
Trends in ORs of binary logistic regression analysis of SSB consumption, standing long jump, and psychological symptoms in Chinese adolescents aged 12–17 years old.

Figure 2 shows the trend of OR values of binary logistic regression analysis of SSB consumption, standing long jump, and psychological symptoms in Chinese adolescents aged 12–17 years. As can be seen from the figure, with the increase in SSB consumption and the decrease in standing long jump performance, the risk of psychological symptoms in adolescents showed an increasing trend, i.e., the OR value showed an overall increasing trend.

Table 5 shows the binary logistic regression analysis of generalized linear models for SSB consumption, standing long jump, and psychological symptoms in Chinese adolescents aged 12–17 years. The binary logistic regression analysis of the generalized linear model was performed with the presence of psychological symptoms as the dependent variable and different combinations of SSB consumption and standing long jump quartile as the independent variables. The model in the generalized linear model binary logistic regression analysis adjusted for age, BMI, family financial income, commuting method to and from school, snacks, and sleep duration. Overall the results of the analysis showed that the risk of psychological symptoms among adolescents in the SSB consumption ≥4 times/week and standing long jump quartile Q1 group was higher than the risk of psychological symptoms among adolescents in the SSB consumption ≤1 times/week and standing long jump quartile Q4 group was 2.05 times (95% CI: 1.76–2.38) higher (*p* < 0.001). When analyzed by gender, the same trend was observed in boys and girls.

**Table 5:** Binary logistic regression analysis of generalized linear models of SSB consumption, standing long jump, and psychological symptoms in Chinese adolescents.

Sex	Classification of interaction		Psychological symptoms	
	SSB consumption	Standing long jump quartile	OR (95% CI)	*P*-value
Boys	≤1 times/week	Q4	1.00	
		Q3	1.04(0.90 ~ 1.20)	0.614
		Q2	1.08(0.90 ~ 1.30)	0.425
		Q1	1.47(1.21 ~ 1.79)	<0.001
	2–3 times/week	Q4	0.98(0.87 ~ 1.11)	0.791
		Q3	1.03(0.91 ~ 1.17)	0.661
		Q2	1.17(1.00 ~ 1.36)	0.054
		Q1	1.23(1.04 ~ 1.46)	0.021
	≥4 times/week	Q4	1.40(1.21 ~ 1.62)	<0.001
		Q3	1.72(1.47 ~ 2.01)	<0.001
		Q2	2.02(1.65 ~ 2.48)	<0.001
		Q1	1.73(1.32 ~ 2.26)	<0.001
Girls	≤1 times/week	Q4	1.00	
		Q3	1.79(1.05 ~ 3.05)	0.032
		Q2	1.95(1.16 ~ 3.30)	0.011
		Q1	2.30(1.36 ~ 3.88)	<0.001
	2–3 times/week	Q4	1.75(0.95 ~ 3.22)	0.071
		Q3	2.19(1.30 ~ 3.72)	<0.001
		Q2	2.18(1.29 ~ 3.67)	<0.001
		Q1	2.37(1.41 ~ 3.99)	<0.001
	≥4 times/week	Q4	3.76(1.84 ~ 7.70)	<0.001
		Q3	2.77(1.59 ~ 4.80)	<0.001
		Q2	3.30(1.93 ~ 5.65)	<0.001
		Q1	4.59(2.69 ~ 7.83)	<0.001
Total	≤1 times/week	Q4	1.00	
		Q3	0.96(0.85 ~ 1.09)	0.548
		Q2	0.95(0.84 ~ 1.08)	0.439
		Q1	1.14(1.01 ~ 1.29)	0.031
	2–3 times/week	Q4	1.01(0.90 ~ 1.13)	0.878
		Q3	1.05(0.94 ~ 1.17)	0.420
		Q2	1.07(0.95 ~ 1.2)	0.263
		Q1	1.13(1.01 ~ 1.27)	0.030
	≥4 times/week	Q4	1.46(1.27 ~ 1.68)	<0.001
		Q3	1.61(1.4 ~ 1.86)	<0.001
		Q2	1.73(1.49 ~ 2.00)	<0.001
		Q1	2.05(1.76 ~ 2.38)	<0.001

SSB, sugar-sweetened beverage; OR, Odds Ratio; 95% CI, 95% Confidence Interval; Generalized linear model binary logistic regression analysis in which the model adjusted for age, BMI, family financial income, commuting method to and from school, snacks, and sleep duration.

## 4 Discussion

The prevalence of psychological symptoms among adolescents is increasing globally, posing a serious threat to adolescent health and future adult health (31). Therefore, it is particularly important to prevent and intervene in adolescents’ psychological symptoms. To the best of our knowledge, this study is the first to analyze Chinese adolescents aged 12–17 years old with a nationwide sample of psychological symptoms and to assess their influencing factors. The results of this study showed that the prevalence of psychological symptoms among Chinese adolescents was 21.2%. A survey of American adolescents showed that the prevalence of psychological symptoms among adolescents was 14.3% (32). Another meta-analysis of psychological symptoms in European adolescents showed that the prevalence of psychological symptoms in adolescents was 15.5% (33). It can be seen that the prevalence of psychological symptoms among Chinese adolescents is relatively high, which should attract sufficient attention and concern. The results of this study also showed that the prevalence of psychological symptoms among Chinese adolescent boys was higher than that among Chinese adolescent girls, which is consistent with the findings of related studies (34). A study on adolescents in the United States found that, with the increasing pressure and social responsibility of society on men’s lives, the risk of psychological problems for boys is higher than that for girls, and attention should be paid to preventing and guiding boys in advance (35). Another study of European adolescents also confirmed that the prevalence of psychological symptoms is higher among boys than girls, with serious negative consequences for boys’ mental health and future employment (36). However, it is of concern that the results are not entirely consistent. A survey of British adolescents showed that girls had a higher prevalence of psychological symptoms than boys (37). There are inconsistent findings in this study. The study shows that girls are reluctant to confide in others when they encounter psychological problems, while boys tend to vent their bad emotions and achieve psychological stress relief by exercising with their peers when they encounter psychological problems such as frustration (38). This shows that the difference in the prevalence of psychological symptoms between boys and girls may be closely related to factors such as cultural background, family environment, economic income, dietary behavior, exercise habits, personality traits, etc., and that in-depth investigations and analyses are necessary in the future (39).

The continued increase in SSB consumption has become an important factor affecting adolescent health worldwide (40). Surveys show that SSB consumption continues to increase globally among adolescents and continues to increase between 2000 and 2020, with an average increase in SSB consumption of about 150 mL per day, with serious implications for physical and mental health and a serious disease burden on the country (41). The results of the present study also showed a strong association between SSB consumption and psychological symptoms in Chinese adolescents, with higher rates and risks of psychological symptoms in adolescents with higher SSB consumption. For example, a survey of adolescents in 12 countries around the world showed that adolescents with daily SSB consumption ≥500 mL had a 1.3–1.5-fold higher risk of depression and anxiety symptoms than non-drinkers, which may be related to sugar-induced neuroinflammation, blood glucose fluctuations, and gut flora disruption (42). Another survey of 14-17-year-olds in the UK found that adolescents in the high SSB intake group (>355 mL/day) had a 40% increased risk of developing psychological symptoms, and girls were more sensitive (43). However, this result is not entirely consistent. A survey of U. S. adolescents showed no statistically significant association between adolescent SSB consumption and psychological problems such as depression and anxiety. The reason for this may be due to confounding factors, such as family socioeconomic status, and overall dietary patterns that mask potential associations with (44). In response to this finding, it may be related to the fact that the groups investigated in the different studies were different, and there were large differences between their cultural backgrounds, and dietary behaviors (45). On the other hand, there are some differences in the seasons in which SSB consumption has been investigated in different studies. Studies have confirmed that SSB consumption is particularly affected by climate throughout the year, with adolescents’ SSB consumption 25–30% higher in summer than in winter (46). This variation is also important in influencing the association between adolescent SSB consumption and psychological symptoms. Further, there were some differences in the control of covariates affecting adolescent psychological symptoms among the different studies analyzed (47). This change is also an important factor influencing the association between SSB consumption and psychological symptoms in adolescents. In this study, the relevant covariates affecting Chinese adolescents’ psychological symptoms were adjusted to better analyze the association between SSB consumption and psychological symptoms. This will help to prevent and intervene in Chinese adolescents’ psychological symptoms in the future.

There is a strong association between adolescent muscle strength levels and health, with relatively little research on the correlation with mental health. It was found that with the change of lifestyle and the increasing time of light physical activity, the muscle strength of adolescents showed a tendency to decrease year by year, which posed a serious threat to physical and mental health (48). It can be seen that focusing on the association relationship between adolescents’ muscle strength and psychological symptoms is of great practical significance in promoting adolescents’ psychological health development. The results of this study show that there is a strong association between standing long jump, which reflects adolescents’ muscle strength, and psychological symptoms. With the decline of adolescents’ muscle strength, the proportion of adolescents suffering from psychological symptoms showed an increasing trend. In the present study, the results of further analysis after adjusting for the relevant covariates showed that there was a significant negative correlation between adolescents’ muscular strength and the prevalence of psychological symptoms. A survey of US adolescents also showed that adolescents with lower muscle strength had a 40% higher risk of developing psychological symptoms compared to those with higher muscle strength (49). There are several specific reasons for this: Firstly, adolescents with high muscular strength are often accompanied by a perfect body shape, and this good external image will be important for the development of adolescents’ mental health, which can enhance self-confidence and self-esteem in life (50). Secondly, adolescents with higher muscle strength are often accompanied by longer periods of physical exercise, and at the same time their level of physical activity is also relatively high, and the dopamine, serotonin, and other hormones secreted by the body during exercise play a positive role in the development of mental health, which is of great importance to the development of adolescent mental health (51). Third, adolescence is a period of dramatic changes in hormone levels. Hormones such as testosterone and estrogen have a significant impact on muscle strength and mental status. Testosterone promotes protein synthesis, increases muscle mass, and leads to increased muscle strength (52). It is also associated with psychological traits such as positive mood, self-confidence, and competitiveness. And estrogen also plays an important role in regulating mood and cognitive functioning (53). Thus, balanced hormone levels help maintain muscle strength and a good mental state in adolescents.

The present study further analyzed the effects of the combined effect of SSB consumption and muscle strength on the development of psychological symptoms in adolescents. The results of this study showed that SSB consumption and muscle strength play a combined effect on the occurrence of psychological symptoms in adolescents, i.e., an increase in SSB consumption and a decrease in muscle strength further increase the risk of developing psychological symptoms in adolescents. Studies have confirmed that excessive SSB consumption in adolescents leads to an increase in energy intake, leading to obesity, while an increase in SSB consumption alone leads to a decrease in the body’s nutrient levels, affecting the absorption and utilization of protein and other nutrients, which in turn affects the retention and enhancement of muscular strength, thus affecting the development of adolescent mental health (54). It has also been shown that excessive SSB consumption leads to an increase in the body’s inflammatory response and that the inflammatory response and oxidative stress counteract the positive effects of muscle strength on psychological symptoms, interfere with normal brain function, and increase the risk of psychological symptoms (55).

The present study has certain strengths and weaknesses. Strengths: To the best of our knowledge, this study is the first to use a nationwide survey sample to analyze the associations that exist between SSB consumption, muscle strength, and psychological symptoms in Chinese adolescents aged 12–17 years, which provides a reference for the prevention and intervention of psychological symptoms in adolescents. However, this study has some limitations. First, this study was a cross-sectional investigation, which only allowed us to understand the associations between adolescent SSB consumption, muscle strength, and psychological symptoms, but not the causal associations between them. Future prospective cohort studies are necessary to analyze the causal associations. Second, although certain covariates were included in this study, the factors affecting adolescents’ psychological symptoms were multifaceted, and it is necessary to include more covariates in the future to better analyze the associations between SSB consumption, muscle strength, and psychological symptoms. Finally, the survey in this study was conducted using a questionnaire, and there were some discrepancies between it and the actual. In the future, a more objective assessment of the instrument should be conducted to improve the accuracy of the study results.

## 5 Conclusion

There is an association between SSB consumption, muscle strength, and psychological symptoms in Chinese adolescents aged 12–17 years. Increasing SSB consumption and decreasing muscle strength increase the risk of psychological symptoms in adolescents. Reducing SSB consumption and improving muscle strength may be effective ways to reduce psychological symptoms in adolescents. The effects of SSB consumption and muscle strength should be considered in future prevention and intervention for adolescents with psychosocial symptoms. Reducing the availability of SSB and increasing physical activity levels can promote the improvement of muscle strength, which in turn can promote the development of adolescent mental health.

## Data Availability

The datasets analyzed in this study are not readily available due to the protection and privacy of the participants. The questionnaire data will not be disclosed to the public. If necessary, you can contact the corresponding author. Requests to access these datasets should be directed to madaoli536@126.com.
